# Origins of Combination Therapy for Tuberculosis: Lessons for Future Antimicrobial Development and Application

**DOI:** 10.1128/mBio.01586-16

**Published:** 2017-03-14

**Authors:** Christopher A. Kerantzas, William R. Jacobs

**Affiliations:** a Department of Microbiology and Immunology, Albert Einstein College of Medicine, Bronx, New York, USA; b Howard Hughes Medical Institute, Albert Einstein College of Medicine, Bronx, New York, USA; Harvard School of Public Health; Harvard Medical School

## Abstract

Tuberculosis is a global health problem that causes the death of approximately 1.5 million people worldwide each year (WHO, p. 1–126, *Global Tuberculosis Report*, 2015). Treatment of drug-susceptible tuberculosis requires combination antimicrobial therapy with a minimum of four antimicrobial agents applied over the course of 6 months. The first instance of combination antimicrobial therapy applied to tuberculosis was the joint use of streptomycin and *para*-aminosalicylic acid as documented by the Medical Research Council of the United Kingdom in 1950. These antimicrobial drugs were the product of many decades of investigation into both organism-derived antibiotics and synthetic chemotherapy and were the first agents in those respective categories to show substantial clinical efficacy and widespread use for tuberculosis. The events leading to the discovery and application of these two agents demonstrate that investments in all aspects of research, from basic science to clinical application, are necessary for the continued success of science in finding treatments for human disease. This observation is especially worth considering given the expanded role that combination therapy may play in combating the current rise in resistance to antimicrobial drugs.

## INTRODUCTION

Tuberculosis (TB) is defined by the World Health Organization (WHO) as an infectious disease caused by *Mycobacterium tuberculosis*, most commonly affecting the lungs, and resulting in symptoms such as fever, night sweats, weight loss, and coughing of sputum and/or blood (1). Tuberculosis was declared a Global Emergency by the WHO in 1994 and is currently ranked as the most common cause of death from an infectious disease, causing an estimated 1.5 million deaths in 2015 (1, 2). Current treatment regimens for drug-susceptible tuberculosis typically achieve cure rates of 85% for new cases of tuberculosis and can achieve cure rates as high as 98%; however, even these first-line regimens require the use of four antimicrobial drugs over the course of 6 months (1, 3).

The necessity for multiple drugs in treating tuberculosis is driven by several factors concerning the causative organism *M. tuberculosis*, including but not limited to the general recalcitrance of *M. tuberculosis* with respect to treatment due to its peculiar cellular structure and metabolism; the propensity of *M. tuberculosis* to persist in the face of drug treatment and/or attack by the host immune system; and the tendency of *M. tuberculosis* to develop resistance to drug therapy (4–6). As a result, treatment of tuberculosis has required the combination of several antimicrobial drugs since the first applications of drug therapy to the disease.

The first combination drug therapy for tuberculosis was the product of years of work in two separate but concurrent lines of inquiry. The first line of inquiry was the development of antibiotics from the first antibiotic (penicillin) to the first antibiotic successfully used to treat tuberculosis (streptomycin) (7, 8). The second was the development of antimicrobial chemotherapy from the first synthetic antibacterial drug (the antisyphilis agent arsphenamine [Salvarsan]) to the first synthetic antimicrobial successfully used to treat tuberculosis (*para*-aminosalicylic acid [PAS]) (9, 10). Consequently, the first combination antimicrobial regimen for tuberculosis was comprised of streptomycin and PAS and all future tuberculosis regimens came about as additions to or modifications of this regimen as new drugs were discovered (11, 12). Importantly, the discoveries that led to the first combination therapy and the subsequent successes in treatment of tuberculosis spanned a range from basic soil microbiology to rigorous clinical trials and highlight the value of investing in all aspects of science to advance the treatment of human disease.

For the sake of accuracy, in the following discussion the term “antibiotic” is used to describe antimicrobials that are derived from living organisms, the term “chemotherapy” is used to describe antimicrobials that are chemically synthesized, and the terms “antimicrobials” and “antimicrobial drugs” are used to refer generically to both of those categories.

## HISTORY OF ANTIBIOTIC DEVELOPMENT FROM PENICILLIN TO STREPTOMYCIN

As is well known at this point, antibiotic development began with the serendipitous discovery by Alexander Fleming that a colony of the mold *Penicillium* resembling species *rubrum* (“*rubrum*” denoting red color) exhibited a lytic effect on nearby colonies of Staphylococcus (8). This mold would later be described as being of the species *notatum*, which was then later reclassified as the species *chrysogenum* (13–15). In a rigorous characterization of the lytic effect of this mold, Fleming showed that both the extracts from the mold colonies and the conditioned broth in which the mold had been grown had bacteriostatic and bactericidal properties against *Staphylococcus*, *Streptococcus*, and *Corynebacterium diphtheriae* but not against various Gram-negative organisms such as *Escherichia coli* and *Haemophilus influenzae* (8). Fleming coined the term “penicillin” to describe the active substance in the extracts. In the same report as the initial discovery, penicillin was also demonstrated to be nontoxic by injection of conditioned broth into rabbits intravenously and into mice intraperitoneally and by topical application of conditioned broth onto the skin and conjunctiva of humans (8).

The clinical success of penicillin did not come immediately, however, and it was not until 12 years after its discovery that its potential would begin to be realized. In that time, René Dubos took up the mantle of antibiotic development and discovered tyrothricin in the extracts of an unidentified soil-dwelling bacterium of the *Bacillus* genus (16, 17). Tyrothricin was shown to have activity against Gram-positive cocci but not Gram-negative bacteria, and it could protect white mice from 10,000 to 100,000 times the lethal inoculum of various strains of *Streptococcus* with only 2 mg of crude extract or 5 μg of purified active substance (18). Some 2 years later, the bacterial species producing the antibiotic was identified as *Bacillus brevis* (now *Brevibacillus brevis*) and tyrothricin was shown to be comprised of two antibiotic substances, gramicidin and tyrocidine (19, 20). The former was determined to be responsible for the effects of tyrothricin on Gram-positive bacteria, and the latter had efficacy against Gram-negative bacteria *in vitro* in broth without peptone but was inactive *in vivo* and had more potent toxicity (19).

Fortuitously, the continued work in the field of antibiotic development influenced Ernest Chain and Howard Florey to begin new investigations into the applications of penicillin (13). The studies of Chain and Florey confirmed both the efficacy of penicillin against *Staphylococcus* and *Streptococcus* in mice and the relative lack of toxicity in rats and in cats (13). Critically, a more detailed report from their laboratories followed within a year that described a method of isolating large amounts of penicillin from culture and storing it in a dry, powdered form (14). These advances allowed enough material to be accrued for a clinical trial in humans, which was pursued in the same study (14). In this trial, penicillin was given by intravenous injection or by oral administration in cases ranging from conjunctivitis to urinary tract infections and in all cases was seen to be efficacious (14). That study opened the door for manufacture of penicillin on a large scale, which then led to more-widespread experimentation and use. Soon, the first dose of penicillin was administered in the United States at then-New Haven Hospital (now Yale-New Haven Hospital), and its miraculous effects on a patient’s clinical status were described as “black magic” by a senior consultant on the case (14, 21). Production of penicillin gradually increased to the point of allowing larger-scale clinical trials, including one of 172 patients in the United Kingdom and one of 500 patients in the United States in which the drug was shown to be generally safe and effective when given intravenously (22, 23). Production continued to increase dramatically afterward, reaching over 600 billion units per month in 1945, and the drug was in wide use by the United States Armed Forces during the latter half of World War II (24).

Unfortunately, penicillin seemed to have minimal if any effect on *M. tuberculosis* (14). However, its great success had inspired Selman Waksman at Rutgers University to probe an extensive repository of soil microbes that he had accumulated in his agricultural research for microbes with antibiotic properties. This work first yielded the discovery of actinomycin, a compound from the newly characterized *Actinomyces antibioticus* species that was observed to exert both bacteriostatic and bactericidal effects on mainly Gram-positive bacteria *in vitro* (25). Despite this efficacy, actinomycin was quite toxic *in vivo* and was not noted to exert an effect on *M. tuberculosis* (25). The second product of Waksman’s work was streptothricin, which, in contrast to actinomycin, exerted bacteriostatic and bactericidal effects on predominantly Gram-negative bacteria *in vitro* and initially showed some activity against *Mycobacterium phlei*, a relative of *M. tuberculosis* (26).

Finally, streptomycin was discovered in 1944 through the work of Albert Schatz in Selman Waksman’s laboratory (7). Streptomycin was less effective than streptothricin in treating fungal infections but had notably lesser toxicity in animal models and was 50 times more effective in killing *M. tuberculosis in vitro* (27, 28). A collaboration was quickly struck with H. Corwin Hinshaw and William Feldman at the Mayo Clinic, who had experience testing chemotherapeutic agents in guinea pigs. Initial studies showed a protective effect of streptomycin in guinea pigs infected with *M. tuberculosis*, and small-scale clinical trials were commenced immediately, ultimately demonstrating a similar protective effect in humans (29, 30).

Larger-scale trials, such as those by the Trudeau Institute and the U.S. Veterans Administration, were initiated soon after (31, 32). The latter would ultimately run for over 8 years and would enroll over 7,000 participants with 22 different classifications of tuberculous disease (33–39). Of crucial importance was the determination of the effect of streptomycin on the most prevalent form of the disease, pulmonary tuberculosis. With meningeal tuberculosis or disseminated miliary tuberculosis, in which mortality was at or near 100% without treatment, any improvement in mortality could be attributed to streptomycin; however, there were many different stages of disease in pulmonary tuberculosis that responded differently to treatment in these trials. Furthermore, disease would spontaneously remit in some patients, confounding the effect of streptomycin (32–34, 40–44). To settle the issue, the Medical Research Council (MRC) of the United Kingdom undertook the task of performing the first ever randomized controlled trial (RCT) designed to test the efficacy of streptomycin against pulmonary tuberculosis in adult patients aged 15 to 30, which would allow a statistical comparison between the effects of streptomycin and the gold standard of treatment at the time, bed rest (45, 46). In an interesting twist of fate, the ethical concerns of including a control group were rendered immaterial by the fact that the MRC had only a limited supply of streptomycin (47, 48). Upon the conclusion of the trial, the results were clear. As assessed by lung radiography, streptomycin monotherapy had improved the condition of patients by 51%, compared to 8% in the control group (45). Yet there were also findings that were extremely concerning. Namely, the infections of 20% of the patients recruited to the trial became resistant to streptomycin by 6 months (2 months after cessation of therapy) and vestibular dysfunction was noted in 65% of patients receiving streptomycin (45).

Adding to these concerning findings, bacteriological characterization by Marjorie Pyle of sputum samples from patients being treated with streptomycin revealed that the proportion of streptomycin-resistant bacilli in a given patient could increase in a linear fashion from a spontaneous resistance background of 1 in 88,750 CFU at the initiation of treatment to 1 in 367 CFU after a mere 5 weeks of treatment (4). A similar investigation by D. A. Mitchison also demonstrated a linear increase in the number of streptomycin-resistant bacilli in patient samples over the course of treatment, even when the total number of bacilli isolated from sputum initially decreased after the commencement of streptomycin therapy (49). Alarmingly, these studies observed bacteria capable of growing in as much as 100 μg/ml and 1 mg/ml of streptomycin, respectively (4, 49). To decrease rates of resistance and to potentially lower the required dose of streptomycin so as to avoid toxicity, it was proposed that another antimicrobial agent should be introduced to the streptomycin regimen. This additional agent would come from the second line of inquiry into antimicrobials, synthetic chemotherapy.

## HISTORY OF CHEMOTHERAPEUTIC DEVELOPMENT FROM ARSPHENAMINE TO PAS

At the turn of the 20th century, chemotherapy was first conceptualized and named by Paul Ehrlich, who had hypothesized that certain chemical agents, when administered to a patient, would travel directly to microbes causing an infection and disrupt them like a “magic bullet” (50). In pursuit of this hypothesis, Ehrlich began screening chemical compounds for efficacy against several microbial pathogens. Early success was had in the discovery of trypan red and atoxyl, synthetic dyes that showed inhibitory activity toward trypanosomes (10). Upon the discovery of *Treponema pallidum* as the causative agent of syphilis, Ehrlich resolved to screen his library of compounds for efficacy against that microbe as well. After testing and retesting sets of compounds, compound “606,” which had previously been set aside, was found to be effective in treating syphilis in a rabbit model of infection in 1907 (10). This compound was later given the generic name arsphenamine and the trade name Salvarsan and was tested rigorously in the clinical setting in over 20,000 patients before initiation of commercial production and marketing. Arsphenamine (Salvarsan) was thus the first successful chemotherapeutic antibacterial agent (10).

Two decades later, while penicillin was still being explored through the application of crude extracts to cutaneous infections, researchers at the Bayer laboratories in Germany were aware of Ehrlich’s prior success and had determined to pursue similar investigations (51, 52). As a direct consequence of those efforts, Gerhard Domagk and colleagues began a search for synthesized compounds that would kill or inhibit the growth of bacterial specimens. Their efforts came to fruition with the discovery of Prontosil, the first sulfonamide antimicrobial, in 1935 (53). In a model of streptococcal sepsis in mice and rabbits that normally resulted in death within 24 to 48 h, Prontosil was able to reduce damage to all tissues and to increase survival in all treated groups; the medication was even effective against osteomyelitis and endocarditis in affected rabbits (53). Trials in humans showed similar efficacy in cases of erysipelas and in a case of sepsis subsequent to a terminated pregnancy (54). In all cases in animals and humans, the drug was shown to have minimal side effects. Following the discovery of Prontosil, several other compounds in the sulfonamide class were shown to have antimicrobial activity, including some that exhibited efficacy against tuberculosis; sulfanilamide, azosulfamide, and sulfapyridine all showed a reduction in the rate of progression of disease in animals infected with *M. tuberculosis* (55). Moreover, a marked reduction of lesions within affected tissues was observed upon histological analysis of treated animals (55). One such sulfonamide, glucosulfone (Promin), was shown by Hinshaw and Feldman to prevent the development of tuberculosis in experimentally infected guinea pigs and, furthermore, to prevent the formation of any gross lesions in 40% of guinea pigs with preexisting infection (55). However, the drug caused hemolytic anemia when given orally (the preferred route of administration) and so further investigations into its use for tuberculosis were tabled upon the discovery of streptomycin, though it later found application in the treatment of leprosy. Fortuitously, the laboratory infrastructure and methodology that Hinshaw and Feldman developed in their trials of Promin primed them for their collaborations with Selman Waksman so that preclinical testing of streptomycin could begin almost immediately upon receipt of materials.

Though Promin had been tabled, the possibility of using chemotherapy to treat tuberculosis inspired Jörgen Lehmann at the pharmaceutical company Ferrosan to pursue this potential avenue of treatment. Lehman read that the addition of salicylate to tuberculosis suspended in phosphate buffer resulted in an increase in oxygen consumption by the bacillus, suggesting the use of salicylate in a potentially important bacterial metabolic pathway (51, 56). As a result, Lehmann hypothesized that a chemically modified salicylate might inhibit the growth of *M. tuberculosis*, essentially as an analog inhibitor of whatever pathway was involved in salicylate metabolism (51). Consequently, in what may be the first example of rational drug design, Lehmann screened over 50 derivatives of benzoic acid to find PAS. Though most of Lehmann’s work is contained in the internal publications of Ferrosan’s journal *Observanda*, Lehmann did publish the findings from two human cases of tuberculosis in an academic journal that showed PAS to be effective in reducing fever and safe when administered orally (9, 51). After more exploratory efforts yielded an improved method of synthesis for PAS, enough material was gathered for further clinical trials. PAS was shown to be effective in reducing or eliminating symptoms and signs of disease in renal tuberculosis, tuberculous meningitis, and tuberculous enteritis (57). Further studies for treatment of pulmonary tuberculosis also showed a marked effect on progression of the disease (58).

## THE FIRST COMBINED ANTIMICROBIAL REGIMEN

Given the complications observed in treatment with streptomycin and the efficacy observed for both streptomycin and PAS individually, the MRC decided to extend their first RCT to include the first combination antimicrobial regimen using both of these agents. In this new trial, the MRC found that, in contrast to streptomycin monotherapy, which yielded streptomycin resistance in 70% of cases after 120 days, combination therapy yielded streptomycin resistance in at most 9% of cases and in 0% of cases in regimens with intermittent streptomycin administration every 3 days (12, 59). That study was then extended yet again to determine which dosage of PAS was optimal for both treatment and prevention of streptomycin resistance. It was found that while 5 g of PAS given daily was sufficient for clinical efficacy compared to 20 g of PAS given daily, results from 41% of subjects showed streptomycin resistance in the former regimen compared to only 7% for subjects in the latter regimen after 5 months of treatment (60–62). Of note, all three trials conducted by the MRC shared the same design, allowing the results of the trials to be compared directly and to be aggregated (60).

## THE PATH TO MODERN ANTIMICROBIAL THERAPY FOR TUBERCULOSIS

With the initial regimen and precedent for how to conduct trials in place, incorporation of new therapies proceeded readily as they were discovered. The many trials and decades’ worth of work have been thoroughly summarized by Wallace Fox, formerly of the MRC (11). A brief summary follows, with a focus on only first-line antimicrobials. With the finding that the hydrazine anticancer agent isoniazid was effective at treating tuberculosis, the drug was quickly moved into clinical trials, where it was shown to be safe and effective but resulted in resistance in 70% of cases after 3 months (63). The combination of isoniazid, streptomycin, and PAS, however, was considered the first curative regimen and had relapse rates as low as 4% in cohorts treated for 1 to 2 years (11). A primary contributing factor to the success of this “triple therapy” regimen was the unlikelihood of developing resistance to all three agents, an event that would occur in only 1 in 10^15^ bacilli, using the rate of spontaneous resistance to streptomycin of 1 in 10^5^ as a reference (4). This estimation is contextualized by the fact that the bacillary burden in cases of severe, cavitary tuberculosis has been estimated to be on the order of 10^8^ to 10^10^ bacilli, making it improbable that a patient would harbor such a multiresistant strain *a priori* (64). The triple therapy regimen was also effective in ensuring that combination therapy with two drugs was still being applied to individuals with primary resistance to any one of the individual drugs in the regimen; at the time that triple therapy was begun, the prevalence of primary resistance for each of the drugs in the general population ranged from approximately 1 to 3% (11). Pyrazinamide, discovered in 1952, was also incorporated into various regimens and was found to provide the shortest duration of treatment for sputum conversion in combination with isoniazid but also the highest rates of toxicity (65). Ethambutol was discovered in 1961 by random screening of compounds against *M. tuberculosis in vitro* and was shown to be more potent than streptomycin in treating tuberculosis in mice (66). Finally, rifamycin was discovered in 1957 but did not see clinical use until the late 1960s as the modified compound rifampin, which was designed for oral delivery. Studies showed that it was relatively nontoxic and was comparably efficacious when used to replace other drugs in combination regimens; most importantly, it showed efficacy in patients whose infections had become resistant to 7 or more other antimicrobial drugs at the time (67).

Early combination therapies were geared toward reducing resistance to one or more of the drugs being used, but ease of administration eventually took precedence as a bridge to ambulatory therapy (11). Simplified, less-frequent dosing schedules were explored, and streptomycin, which required injections every 3 days, was replaced with other drugs that could be administered orally (11). Direct observation was then incorporated to decrease the risk of developing resistance by ensuring compliance with the drug regimen for the entire duration of treatment (11). Finally, treatment duration was itself explored and a regimen with an initiation phase of a combination of isoniazid, rifampin, and pyrazinamide and a continuation phase of isoniazid and rifampin showed that 6 months of treatment was equivalent to 9 months of treatment, thus establishing the basis of the modern antimicrobial regimen for tuberculosis (68).

Yet despite the many successes of combination therapy, drug resistance has continued to grow. In 2014, the number of cases of active tuberculosis with resistance to rifampin, isoniazid, and at least 1 second-line medication (also known as multidrug-resistant tuberculosis [MDR-TB]) reached an estimated 450,000 (1). Moreover, whereas almost all cases of MDR-TB were attributed previously to acquired resistance as a resulted of failed therapy within an individual, there is now evidence that a substantial number of cases of MDR-TB are the result of transmission of resistant organisms from one person to another (69). This increased prevalence of resistance in the face of multidrug therapy can be attributed at least in part to the imperfection of the current regimen, whose 6-month duration results in issues of medication compliance and affords *M. tuberculosis* the opportunity to engender resistance even in the face of overwhelming odds. To further improve the combination therapy for tuberculosis and combat the rising tide of resistance, continued and increased investment in drug discovery, preclinical models, and clinical regimen optimization may hold the determinants of success. Interestingly, the history of combination therapy itself provides insight on some approaches to advance each of these critical endeavors.

## LESSONS FROM THE HISTORY OF COMBINATION THERAPY

Clearly, efforts in drug discovery have the potential to add new, more efficacious agents to current regimens with the goal of shortening treatment, increasing cure rates, and decreasing resistance. In terms of lessons from history that may be applicable to this area, there are several. The development of combination therapy through the use of natural product isolation, synthetic chemistry, and rational drug design provides great substrate for future approaches to discovery. Aside from the more general inference that continuing to pursue these drug discovery modalities may yet yield important discoveries, there may also be fruit more specifically in revisiting soil microbiology, an endeavor already under way at certain institutions (70, 71). New approaches and technologies may allow the isolation of new antimicrobial compounds from microbes that were first identified long ago or that have remained unculturable, perhaps even in a high-throughput fashion (72, 73). It is worth considering that there may likewise be many synthetic drug candidates that, though created in the distant past, remain untested in their capacity to inhibit the growth of *M. tuberculosis* specifically (74).

Another key to successful drug discovery efforts is the selection and use of appropriate preclinical models. Certainly, if Hinshaw and Feldman had not been already conducting their work in the guinea pig model of tuberculosis, the translation of streptomycin to the clinic might have been delayed substantially. The use of multiple preclinical species such as mice, rats, rabbits, and guinea pigs in those historical studies is also worthy of remark, with certain antimicrobial agents showing variable exposure and efficacy in different species even then (29, 75–77). While analysis of parameters of new drugs in at least two preclinical species is standard in the field of pharmacokinetics, efficacy is often validated in only one, perhaps as a result of the difficulties, expense, and effort of recapitulating disease in multiple species. However, differences among species may provide insights into unrecognized aspects of drug mechanisms, may aid prediction of efficacy in humans, and could further our knowledge of the preclinical models themselves, facilitating rational preclinical model selection in the future.

Regimen optimization involves the incorporation of new therapeutics into treatment regimens but also includes revisiting the use of preexisting therapeutics as well as the dosage, timing, and duration of all of the agents involved. In this regard, the history of combination therapy shows that while tremendous efforts were brought to bear and important strides were made, the possibilities of each of these variables were by no means exhausted. Indeed, the prospect of attempting to systematically evaluate each existing agent across all of these variables and in all combinations seems even now an impossible task; but while undertaking investigation seems daunting, these unexplored variations mean that there is great potential for finding improvements. Furthermore, each permutation of therapy need not be explored, as a rational approach incorporating the understanding of pharmacology and the data generated through many years of rigorous trials may aid in guiding these efforts. In this endeavor, the MRC may also serve as an example in constructing trials that are comparable to one another, so that subtle improvements in regimen outcome may be more easily sieved from the masses of data generated. In addition, new methodologies may be used, perhaps including a high-throughput drug-screening approach in which cocktails of drugs are tested *in vitro* or in preclinical models *en bloc* with later investigation into the issue of which agents in the mixture contribute to efficacy.

Notably, the work of regimen optimization is already under way. Characterization of the enzymatic properties of the beta-lactamase of *M. tuberculosis* recently led to the discovery that carbapenem antibiotics combined with clavulanic acid can be used to kill drug-resistant *M. tuberculosis in vitro*, and clinical trials have commenced to test the efficacy of this combination in humans (78, 79). A revisitation of the antimicrobial clofazimine has shown that incorporation of this drug into a regimen for MDR-TB led to a shortening of the time to cure to 5 months instead of greater than 9 months in mice (80). Other investigations have now demonstrated that clinical doses of certain tuberculosis medications fall into a dose range known as a “mutant selection window,” preferentially selecting for resistance by killing susceptible organisms but not obtaining a high enough exposure to limit levels of preexisting resistant mutants to any appreciable degree; this work has prompted the reevaluation of dosing of the antimicrobial drugs in question (81, 82). In a similar vein, trials to reassess the dosage of rifampin are also currently in progress (83).

More broadly speaking, the paths of research that led to the discovery of both antibiotics and chemotherapeutic agents serve as reminders that investments into basic science are vital—the microbiology that resulted in penicillin and streptomycin was not originally intended for the purpose of developing therapeutics. This same observation also underscores that paradigm-shifting discoveries often come from unexpected places. In turn, while pursuing the now well-established methods of therapeutic discovery, it is worth remaining open to therapies or applications that have yet to play a role in tuberculosis therapy. For instance, phage therapy, therapeutic vaccines, immunomodulators, or biologics such as antibodies may come to contribute to future regimens. Some of these modalities may prove to be too cost-prohibitive for drug-susceptible tuberculosis; however, they may still find a use in the treatment of drug-resistant tuberculosis, for which costs are already quite high. It is also worth noting that basic science investigations into bacterial persistence have uncovered the importance of this phenomenon in facilitating the development of bacterial resistance and have identified the related pathways as highly promising targets for new therapeutic development (84, 85). In bringing to the clinic whatever new treatments may be discovered, the historical collaboration between academia and industry that resulted in the vast scale of streptomycin production can also serve as an example, and similar complementary partnerships may be valuable moving forward.

These lessons are applicable beyond treatment of tuberculosis. With the advent of highly active antiretroviral therapy (HAART) for HIV and curative multidrug therapy for hepatitis C, there are further examples of the application of combination therapy for the treatment of intractable infectious diseases (86, 87). Certain insights gleaned from tuberculosis history may be less transferrable to these multidrug therapies; however, others are quite pertinent. For example, the use of multiple preclinical species to evaluate drug efficacy would be difficult in the case of HIV, while continued regimen optimization could provide important improvements to the already effective HAART and hepatitis C treatments. In fact, even the current monotherapy for infectious agents such as staphylococci may be improved upon, and initial combination therapy may help limit the rise and spread of resistance. In these musings, it becomes apparent that perhaps the most important lesson from the history of combination therapy for tuberculosis is that resistance seems to be the ultimate and natural consequence of the selective pressures of antimicrobial drugs and that continued innovation is necessary even with the existence of seemingly effective treatment. In essence, there is no holding ground in combating drug resistance; there is only gaining ground or losing it.

## CONCLUDING REMARKS

After the overcoming of several failures, combination therapy for tuberculosis was brought to clinical validation with the results of the MRC randomized controlled trial published in 1950 (12, 59). The results and protocols established in that study, along with those from concurrent studies in the United States, set the foundation on which the drug discovery and regimen optimization for tuberculosis were built in the next 50 years. These laborious efforts, in turn, yielded the standardized regimen now widely practiced as part of the modern antituberculosis strategy known as “directly observed treatment, short-course,” or DOTS, which has been shown in some regions to be able to cure as many as 98% of drug-susceptible cases (1, 3, 88, 89). These historical studies highlight the steady progress of science in confronting challenging biomedical problems. However, the work to cure tuberculosis is not done, and increasing resistance threatens to undo much of the progress that has been made. To combat this growing problem, it is worth turning to the lessons learned over the many decades of work in basic drug discovery, preclinical research, and clinical trials that led to the initial achievements in tuberculosis therapy. It is vital to continue to support these endeavors to ensure future successes in reducing the global burden of tuberculosis and other diseases that utilize combination therapy, now and in the future.
